# Accuracy of Budget Impact Projections in Bulgarian Health Technology Assessment: A Five-Year Validation Study (2020–2025)

**DOI:** 10.3390/healthcare13222990

**Published:** 2025-11-20

**Authors:** Kostadin Kostadinov, Ralitsa Raycheva, Iva Zdravkova-Aneva, Margarita Shopova, Evgeni Ovchinnikov, Plamen Petkov

**Affiliations:** 1 Department of Social Medicine and Public Health, Faculty of Public Health, Medical University of Plovdiv, 4002 Plovdiv, Bulgaria; r.raycheva@mu-plovdiv.bg (R.R.); iva.zdravkova-aneva@mu-plovdiv.bg (I.Z.-A.); 2 Environmental Health Division, Research Institute at Medical University of Plovdiv, 15-A “Vasil Aprilov” Blvd., 4002 Plovdiv, Bulgaria; 3 Department of Statistics and Applied Mathematics, Tsenov Academy of Economics, 2 Em. Chakarov St., 5250 Svishtov, Bulgaria; m.shopova@uni-svishtov.bg (M.S.); e.ovchinnikov@uni-svishtov.bg (E.O.); p.petkov@uni-svishtov.bg (P.P.)

**Keywords:** health technology assessment, budget impact analysis, reimbursement, Bulgaria, patient uptake, expenditure forecasting, oncology, immunology, national health insurance fund, real-world

## Abstract

**Highlights:**

**What are the main findings?**

- Health Technology Assessment (HTA) budget projections in Bulgaria systematically underestimated patient uptake for medicines approved in 2019, with realized volumes exceeding forecasts by a median factor of 1.6 by Year 5, driven primarily by oncology and immunology products (Bavencio, Skyrizi, Tremfya).
- Expenditure deviations were predominantly volume-driven rather than price-driven, with immunology contributing +€17.4 M and oncology +€5.0 M in budget overshoots, while overall cost elasticity remained near-proportional (β = 1.002), masking substantial product-specific heterogeneity.

**What are the implications of the main findings?**

- Bulgaria’s HTA system requires strengthened epidemiological infrastructure—including national disease registries and real-world data integration—to improve forecasting accuracy and enable dynamic, indication-responsive budget impact models that adapt to evolving clinical use.
- Policymakers should implement adaptive contracting mechanisms (e.g., managed-entry agreements, outcome-based pricing) and continuous post-launch surveillance to better align budget planning with actual utilization patterns, ensuring fiscal sustainability without compromising patient access to innovative therapies.

**Abstract:**

**Background:** Budget Impact Analysis is an integral part of the Health Technology Assessment in Bulgaria, informing reimbursement decisions of the National Health Insurance Fund. Inaccurate projections risk both fiscal unsustainability and restricted patient access to innovation. Yet projection accuracy methods remains uncertain, particularly given limited epidemiologic data and evolving clinical use. **Objectives:** This study aimed to assess the empirical validity of Health Technology Assessment budget-impact projections for medicines approved in 2019 by comparing projected patient volumes and expenditures with real-world National Health Insurance Fund reimbursements through 2025, and to identify drivers of divergence across therapeutic areas and reimbursement channels. **Methods:** We conducted a retrospective cohort analysis linking 2019 Health Technology Assessment submissions with monthly National Health Insurance Fund claims for both hospital and outpatient reimbursement channels. Actual utilization was calculated as the annualized median number of treated patients per month. Projected costs were derived by multiplying HTA-projected patient volumes by the observed unit cost per patient-month. We quantified deviations using observed-to-projected ratios and absolute gaps and assessed the relationship between projected and actual expenditures using a log–log regression model. **Results:** By September 2025, realized volumes typically exceeded projections (median ratio 1.6; range 0.02–21.3). Large overshoots were observed for Avelumab, Risankizumab, and Guselkumab; Cobimetinib and Abemaciclib remained below forecast. Expenditure deviations were driven predominantly by volume: immunology (+€17.4 million) and oncology (+€5.0 million) accounted for the largest absolute gaps. Elasticity was near proportional overall (β = 1.002; standard error = 0.24; R^2^ = 0.50), lower in hospitals (β = 0.79; *p* = 0.055) and higher in outpatient care (β = 1.30; *p* = 0.003). **Conclusions:** Health Technology Assessment Budget Impact Analyses captured broad cost scaling but systematically missed product-specific uptake, with deviations largely volume-driven. Strengthening national registries and real-world data pipelines, and adopting dynamic, indication-responsive contracting and forecasting, could materially improve budget predictability while preserving access to innovation.

## 1. Introduction

Budget Impact Analysis (BIA) estimates the financial consequences of adopting a health intervention for a budget holder (such as a national payer or health system) over a short to medium time horizon, typically one to five years [1]. Unlike cost-effectiveness analysis, which evaluates value for money, BIA focuses on affordability and the resources required for implementation [2,3,4]. By translating clinical and economic evidence into fiscal projections, BIA estimates patient numbers and expected expenditures under real-world conditions to inform payer decisions and support balanced resource allocation [5,6,7,8].

To promote methodological consistency across Europe, the International Society for Pharmacoeconomics and Outcomes Research (ISPOR) provides widely adopted guidance on how BIAs should be designed and reported [1,2,3,4]. Despite these guidelines, many published BIAs still face recurrent methodological weaknesses [9,10]. Common issues include limited transparency of inputs, insufficient justification for epidemiological assumptions (incidence, prevalence, and eligibility) [11], inadequate sensitivity analyses to test robustness, and a lack of validation against real-world evidence (RWE). Such gaps can produce inaccurate forecasts, creating risks of budget shortfalls or inefficient allocation of resources [1,2,9,10,11].

Reliable epidemiological inputs, covering incidence, prevalence, and the eligible patient population, are fundamental to credible BIAs. Underestimating the target population can generate budget pressures and restrict access, whereas overestimation can misallocate funds away from other priorities [2,3]. Robust data are therefore essential for sound resource use and cost estimates in both baseline and future scenarios [1,3,11].

Integrating RWD has become critical to improving both the accuracy and policy relevance of BIA and Health Technology Assessment (HTA). Unlike models based solely on trial evidence or expert opinion, RWD captures actual uptake, adherence, discontinuation, and switching, thereby reducing forecasting error and aligning projections more closely with observed practice [12,13,14].

Within the European Union, the European Health Data Space (EHDS) is expanding secure, cross-border access to data for research and policy. Best practice recommends sourcing epidemiological inputs from administrative claims, electronic health records, and disease registries, with transparent reporting and adjustments for underreporting or multimorbidity. Validation should follow ISPOR guidance, combine local expert review with scenario-based sensitivity analyses, and adhere to FAIR (Findable, Accessible, Interoperable, Reusable) principles to strengthen transparency, reproducibility, and comparability [1,2,15,16,17].

Accuracy is especially important in vulnerable or data-sparse populations—such as rare diseases, pediatric or geriatric groups, and socioeconomically disadvantaged communities—where underrepresentation in registries or administrative data can mask true burden and unmet need, exacerbating inequities in access and funding decisions [18,19,20].

In Bulgaria, HTA is mandatory for inclusion in the Positive Drug List (PDL), enabling reimbursement by the National Health Insurance Fund (NHIF). The system is governed by Ministerial Order No. 9 (2015) and the Medicinal Products in Human Medicine Act (2019). The National Council on Prices and Reimbursement of Medicinal Products (NCPRMP) applies external reference pricing (based on the lowest price among selected European Union comparators), mandatory discounts, and multi-criteria HTA evaluations that include clinical, cost-effectiveness, and budget impact components [21].

Reimbursed medicines are funded through two main channels. In the outpatient pharmacy channel, the only public insurance company—NHIF—reimburses pharmacies directly for medicines dispensed to ambulatory patients, covering full or partial retail prices. In the hospital channel, the NHIF funds public and private hospitals for high-cost or hospital-administered medicines (e.g., oncology or hematology), in addition to standard payments for clinical pathways. These distinct pathways complicate forecasting because uptake dynamics and procurement mechanisms differ materially between settings [21,22].

Forecasting is further challenged by Bulgaria’s data-limited environment, marked by incomplete epidemiology, fragmented registries, and heterogeneous coding practices [23]. Budget impact models often depend on assumptions about eligibility, treatment duration, market substitution, and adoption rates that may change rapidly after launch. Additional uncertainty arises from evolving clinical guidelines, procurement cycles, hospital budget ceilings, co-payment levels, regional disparities, and prescribing norms [24,25].

Moreover, Bulgaria’s HTA challenges reflect broader patterns observed across Central and Eastern Europe, yet with distinct intensities. While Poland, Hungary, and the Czech Republic have established national health technology assessment agencies with standardized BIA methodologies, Bulgaria’s NCPR operates with more limited institutional capacity and data infrastructure [24]. Compared to Hungary’s comprehensive claims linkage systems and Poland’s mandatory pharmaceutical registry reporting, Bulgaria lacks integrated disease registries and longitudinal patient tracking capabilities, resulting in greater reliance on manufacturer-submitted epidemiological assumptions that often prove inaccurate in practice [23].

The rapid diffusion of high-cost biologics and immunotherapies in EU and Bulgaria highlights the need for adaptive forecasting. Static, one-off projections become quickly outdated as indications expand and treatment pathways evolve [25,26]. Embedding real-world utilization into adaptive models, and linking reimbursement mechanisms (e.g., indication-based pricing, outcome-based contracts, or managed-entry agreements) to observed uptake—can strengthen fiscal control while protecting patient access [27,28,29,30,31,32,33].

These forecasting challenges occur against a broader backdrop of complexity in pharmaceutical access and reimbursement across health systems. Recent evidence highlights the influence of structural inequities in access to medicines, transportation and geographic barriers to care, and variation in how HTA frameworks are applied across countries [34,35,36]. In parallel, emerging research on digital health technologies and their economic evaluation [37] illustrates how the rapidly evolving therapeutic and technological landscape further increases uncertainty. Together, these studies reinforce the need for robust, real-world-informed methodologies that can better anticipate utilization patterns and support more reliable budget planning.

Accordingly, this study evaluates the accuracy of HTA budget impact projections in Bulgaria by comparing projected patient numbers and expenditures for medicines approved in 2019 with realized NHIF reimbursement data from 2020 to 2025. By analyzing discrepancies across therapeutic areas and reimbursement channels, we try to identify key drivers of forecasting error, including indication expansion, administrative delays, and competitive dynamics, and outline alternative policy measures to strengthen the HTA framework, enhance epidemiological data infrastructure, and support sustainable access to innovative therapies. As these manufacturer-submitted projections directly inform reimbursement inclusion and pricing decisions by the NCPR, assessing the real-world validity is also essential for improving the robustness and accountability of reimbursement governance.

## 2. Materials and Methods

### 2.1. Study Design and Data Sources

This study employed a retrospective, observational, and comparative design aimed at evaluating the empirical validity HTA budget impact projections in Bulgaria. The analysis compared the projected number of treated patients stated in HTA reports and their associated estimated expenditures with real-world reimbursement data derived from the NHIF.

The reference study cohort consisted of all medicinal products that received a positive HTA decision in 2019 from the NCPR. Full HTA dossiers are not publicly accessible in Bulgaria; however, official abstracts summarizing positive decisions are available online on the NCPR website (https://www.ncpr.bg/en/positive-decision-to-include-a-medicinal-product-belonging-to-a-new-international-2019_en.html accessed 24 October 2025). These abstracts contain the projected number of patients for the first- and fifth years following market entry, as required by Article 25 (1) of the Bulgarian Ordinance on HTA. Projections for 2020 (Year 1) and 2025 (Year 5) were treated as baseline and follow-up reference years, respectively. Data were available for all assessed products except Nusinersen (Spinraza^®^), for which patient estimates were not published. This medicine report included estimated frequencies (consistence with incidence) but lacked explicit patient counts.

Observed (real-world) data on reimbursed patient counts and public expenditure were obtained from administrative datasets compiled from NHIF claims. Two complementary open-access datasets were used. Both datasets are derived from official NHIF administrative reporting streams, validated and restructured for research use. The first, “Expenditures and Patient Counts for Antineoplastic and Coagulopathy Medicines in Hospital Treatment” [38], covers hospital-based reimbursements for high-cost medicines outside clinical pathways. The second, “Expenditures and Patient Counts for Home Treatment Medicines and Medical Products 2021–2025” [39], contains pharmacy-level data for outpatient reimbursement of home treatment medicines across all NHIF regional offices. Both datasets include monthly records of reimbursed costs (in BGN), patient counts, ATC classification, ICD-10 diagnoses, and product characteristics.

For both datasets, data cleaning and harmonization procedures were performed to ensure consistency across reporting sources. All text variables were converted to lowercase, white spaces were trimmed, and inconsistent separators were standardized. Monetary values were converted from BGN to EUR using the fixed exchange rate of 1 EUR = 1.95583 BGN. Hospital data reflect NHIF payments to hospitals that purchase medicines either through public procurement procedures for public hospitals or directly for private providers reimbursed by NHIF. Outpatient data represent NHIF payments to community pharmacies for medicines dispensed to patients in ambulatory settings.

### 2.2. Analytical Framework

HTA projections were treated as expected uptake in the first and fifth post-assessment years (2020 and 2025). Patient volumes were linearly interpolated between the start year (2020) and end year (2025) projections from each HTA submission. Real-world utilization was derived by aggregating monthly data at the product level, calculating the median monthly patient count across all available months within each year. To account for incomplete calendar years, we tracked the actual number of months observed per product-year (ranging from 1 to 12 months). Unit costs per patient-month were calculated from observed data as the median monthly total cost divided by the median monthly patient count. These observed unit costs were applied to HTA-projected patient volumes (multiplied by the number of observed months) to estimate a “Projected Cost Scenario,” which was contrasted with the “Actual Cost Scenario” derived directly from NHIF data (sum of all monthly costs). For each product and year, we computed the difference between actual and projected costs (gap cost) and the ratio of actual to projected costs (actual-to-projected cost ratio; i.e., actual divided by projected). These metrics were summarized by funding channels (hospital and outpatient) and by year (2020 and 2025).

### 2.3. Statistical Analysis

All statistical analyses were conducted in R (Vienna, Austria, version 4.5.1, [40]) using the tidyverse [41] package family. Descriptive analyses were used to summarize projected and actual numbers of patients, expenditures, and ratios of observed to projected costs. The main analytical model quantified the relationship between projections and realized spending using a log–log linear regression (i.e., a linear model on natural log-transformed costs) at the product level for 2025. The regression model was specified as follows:

ln(Actual Cost)=α+β×ln(Projected Cost)+ε

where β represents the elasticity of actual relative to projected expenditure. The coefficient β was interpreted as the percentage change in actual costs associated with a 1% change in projected costs. Values of β below 1 indicate that actual expenditures increase less than proportionally with projected spending; for example, β = 0.7 implies that actual costs rise by 0.7% for every 1% increase in projected cost. Values of β above 1 indicate that projections systematically underestimate costs for higher-spending products, while β near 1 suggests proportional scaling. From a policy perspective, elasticity near 1 indicates that HTA forecasting methods are broadly reliable for budget planning across different expenditure levels, whereas systematic deviations (β ≠ 1) signal the need for methodological adjustments—such as improved epidemiological inputs or dynamic modeling—to prevent budget shortfalls or inefficient resource allocation.

Model fit was assessed using the coefficient of determination (R^2^) and statistical significance of coefficients was evaluated using standard errors and *p*-values from the regression output. Coefficients were considered statistically significant when *p* < 0.05. The analysis included all products with complete cost data for 2025 (both projected and actual costs greater than zero). Additional stratified analyses were performed by funding channel (hospital versus outpatient pharmacy) to assess whether elasticity patterns differed between reimbursement settings. Sensitivity analyses could be performed by excluding products with incomplete data or extreme deviations (ratios exceeding tenfold differences) to ensure robustness of estimates.

## 3. Results

### 3.1. Medicine Cohort Characteristics

In 2019, twelve medicinal products received HTA decisions in Bulgaria, serving as the baseline for this analysis (Table 1). The HTA reports included budget impact projections for the first to fifth years post-assessment (2020–2025). These medicines addressed a wide range of therapeutic indications, with oncology being the most prevalent category. Six of the twelve products (50%) targeted malignant diseases: Alunbrig^®^ (Brigatinib), Bavencio^®^ (Avelumab), Cotellic^®^ (Cobimetinib), Imfinzi^®^ (Durvalumab), Imnovid^®^ (Pomalidomide), and Verzenios^®^ (Abemaciclib). The remaining six products were indicated for hematology (Hemlibra^®^, Emicizumab), infectious diseases (Juluca^®^, Dolutegravir/Rilpivirine), psychiatry (Latuda^®^, Lurasidone), immunology and dermatology (Skyrizi^®^, Risankizumab; Tremfya^®^, Guselkumab), and neurology (Spinraza^®^, Nusinersen).

Analysis of reimbursement datasets revealed that eleven of the twelve medicines (91.7%) approved in 2019 were included in at least one reimbursement channel by 2025. Five medicines (41.7%) were reimbursed through the outpatient channel, primarily for chronic or ambulatory conditions such as hemophilia, schizophrenia, psoriasis, and spinal muscular atrophy. These included Hemlibra^®^ (Emicizumab), Latuda^®^ (Lurasidone), Skyrizi^®^ (Risankizumab), Spinraza^®^ (Nusinersen), and Tremfya^®^ (Guselkumab). Six products (50%) were reimbursed via the hospital channel, corresponding to high-cost oncological or hematological therapies typically administered in inpatient settings: Alunbrig^®^ (Brigatinib), Bavencio^®^ (Avelumab), Cotellic^®^ (Cobimetinib), Imfinzi^®^ (Durvalumab), Imnovid^®^ (Pomalidomide), and Verzenios^®^ (Abemaciclib). Juluca^®^ (Dolutegravir/Rilpivirine) was not included in either reimbursement channel, as antiretroviral therapy for HIV is funded through a separate national program outside the National Health Insurance Fund (NHIF) framework.

### 3.2. Patient Volumes Relative to Projections

Patient uptake for the 2019 HTA cohort significantly exceeded projections for most medicines, with discrepancies becoming more pronounced by the fifth year (2025) (Table 2). In the first year (Y1, 2020, except Alunbrig^®^ first year 2021), observed-to-projected patient volume ratios were relatively close to unity (median 1.1, range 0.10–1.25), indicating modest deviations from forecasts. However, by the fifth year (Y5, 2025), actual patient volumes were substantially higher (median ratio 1.6, range 0.02–21.3), reflecting rapid clinical adoption and expanded indications. The median annualized growth in realized patient utilization was approximately 70% per year, compared to a projected 25%, highlighting a significant underestimation in HTA forecasts.

The largest deviations in Y5 were observed for Bavencio^®^ (Avelumab, ratio = 21.3, range 19.4–22.2), Tremfya^®^ (Guselkumab, 1.94, 1.68–2.23), Skyrizi^®^ (Risankizumab, 2.50, 2.13–2.80), and Imfinzi^®^ (Durvalumab, 1.55, 1.48–1.62). These products, primarily in oncology and immunology, experienced accelerated uptake, likely due to expanded clinical indications and broader patient eligibility. For example, Bavencio’s patient numbers grew from a monthly median of 60 in Y1 to 2040 in Y5, far exceeding the projected 8 patients in Y5. In contrast, Cotellic^®^ (Cobimetinib) and Verzenios^®^ (Abemaciclib) were the only products with lower-than-projected utilization. For Cotellic^®^ (Cobimetinib) the patient ratio was 0.02 (range 0.02–0.04), declining from 60 patients in Y1 to 12 in Y5 against a projected 45, possibly due to competition from alternative therapies or niche indications. Other products, such as Hemlibra^®^ (Emicizumab, 0.74, 0.63–0.92) and Latuda^®^ (Lurasidone, 0.97, 0.95–1.01), showed steadier adoption, with ratios close to or slightly below projections, indicating more predictable uptake patterns.

When aggregated by therapeutic class, immunology products (Skyrizi^®^, Tremfya) exhibited the highest Y5 realized-to-projected ratios (mean = 2.22), driven by strong demand for chronic treatments like plaque psoriasis. Oncology products showed a higher but more variable mean ratio (4.25), reflecting significant heterogeneity, with Bavencio^®^ and Imfinzi^®^ outperforming projections while Cotellic^®^ and Verzenios^®^ (0.67, 0.64–0.74) underperformed. Rare-disease therapies (Spinraza^®^, Hemlibra) and psychiatric therapies (Latuda^®^) remained closer to projections (ratios ≈ 0.7–1.0), suggesting more stable adoption patterns.

By reimbursement channel, hospital-administered products (e.g., Bavencio^®^, Imfinzi^®^) exhibited larger absolute volume increases due to their high-cost, specialized nature, while outpatient therapies (e.g., Latuda^®^, Hemlibra) showed steadier but still positive deviations.

### 3.3. Expenditure Relative to Projections

**Figure 1:**
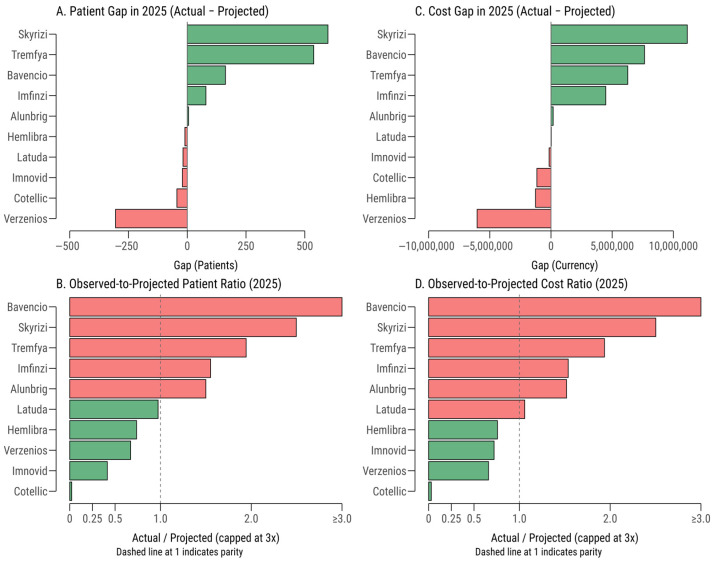
Realized 2025 outcomes versus HTA budget forecasts by medicine. Bar charts show: (**A**) Patient gap (Actual − Projected), (**B**) Patient ratio (Actual/Projected), (**C**) Cost gap in EUR (Actual − Projected), and (**D**) Cost ratio (Actual/Projected). Bars are green when above projections (gap > 0 or ratio > 1) and red when below. Dashed reference lines mark parity (0 for gaps, 1 for ratios). Ratios are truncated at 3× for readability. Patient actuals are annualized medians of monthly treated counts; costs are total NHIF-reimbursed amounts in EUR. Products without budget projections (e.g., Spinraza^®^) are omitted.

Expenditure trends for the 2019 HTA cohort revealed significant deviations from projected budgets, particularly for high-cost hospital-administered medicines, with discrepancies amplifying by the fifth year (2025) (Figure 1). In 2020 (Y1, except Alunbrig 2021), total actual expenditure ranged from 35–45% of projected budgets, with cost ratios (actual/projected expenditure) spanning from 0.10 for Imnovid^®^ (Pomalidomide) to 1.10 for Bavencio^®^ (Avelumab). Outpatient medicines, such as Hemlibra^®^ (Emicizumab, cost ratio = 0.12) and Latuda^®^ (Lurasidone, 0.41), consistently fell below projections, reflecting conservative early uptake and delayed reimbursement penetration in chronic care settings.

By 2025 (Y5), the expenditure landscape shifted markedly, with several products significantly exceeding their projected budgets. Bavencio^®^ (cost ratio = 21.5), Skyrizi^®^ (Risankizumab, 2.50), and Tremfya^®^ (Guselkumab, 1.94) exhibited the largest overshoots, driven by higher-than-expected patient volumes and expanded clinical indications. In absolute terms, immunology products (Skyrizi^®^, Tremfya) contributed the largest budget overshoot (+€17.4 million), followed by oncology (+€5.0 million), with Bavencio^®^ and Imfinzi^®^ (Durvalumab, cost ratio = 1.54) as key drivers in the latter category. Conversely, rare-disease therapies, such as Hemlibra^®^, showed modest underspending (−€1.3 million). Cotellic^®^ (Cobimetinib, cost ratio = 0.03) and Verzenios^®^ (Abemaciclib, 0.66) remained significantly below projections, likely due to niche indications, declining market share, or competition from alternative therapies.

Decomposition analysis indicated that volume effects accounted for 95–100% of cost gaps across most products, with price effects contributing less than 10%. For instance, Bavencio’s €7.6 million overspend in Y5 was almost entirely driven by patient numbers rising from a projected 8 to an actual 2040. Similarly, Skyrizi’s €11.1 million cost gap reflected a surge from 400 projected to 11,976 actual patients. In contrast, Imnovid (Pomalidomide) and Latuda showed mixed volume-price interactions, suggesting additional influences such as pricing adjustments or reimbursement restrictions. Products like Spinraza^®^ (Nusinersen) lacked budget projection data, precluding cost ratio analysis.

By therapeutic class, immunology products exhibited the highest mean cost ratio in Y5 (2.22), followed by oncology (mean = 1.69, highly variable due to Cotellic’s underperformance). Rare-disease and psychiatric therapies (e.g., Latuda^®^, Hemlibra) remained closer to projections (cost ratios ≈ 0.7–1.0). Hospital-administered products accounted for the largest absolute expenditure increases, reflecting their high per-patient costs, while outpatient therapies showed steadier but still positive deviations. These trends underscore the challenges of forecasting budget impacts for innovative therapies with evolving indications and rapid adoption.

### 3.4. Elasticity Analysis

**Figure 2:**
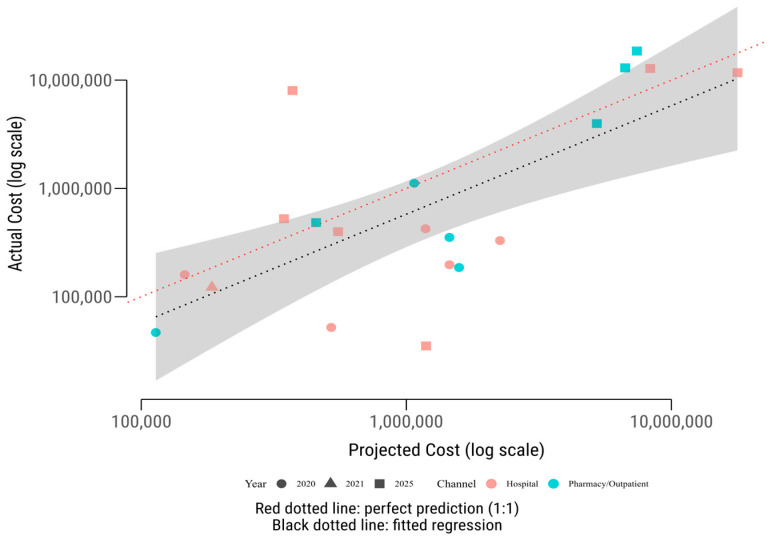
Log–log relationship between projected and actual costs. Scatterplot showing the association between HTA-projected and realized product-level expenditures (EUR, log scale). Points represent medicines by reimbursement channel (color) and year (shape). The red dotted line marks perfect parity (1:1), while the black dotted line shows the fitted regression (elasticity ≈ 1.00, *p* < 0.001).

The elasticity analysis examined the relationship between projected and actual expenditures revealed a near-proportional association with significant product-specific variability (Figure 2). The estimated elasticity coefficient was 1.002 (SE = 0.24, *p* < 0.001), indicating that a 1% increase in projected HTA expenditure was associated with an approximately 1.02% increase in actual expenditure. This near-unity elasticity suggests that, on average, products with higher forecasted budgets incurred proportionally higher actual costs, though substantial deviations persisted.

The regression model explained approximately 50% of the variance in actual expenditures (R^2^ = 0.50), with a residual standard deviation of 1.48 log-points, highlighting considerable heterogeneity across products. The non-significant intercept (β_0_ = −0.58, *p* = 0.86) indicates no consistent systematic bias in HTA projections when controlling for expenditure magnitude, meaning projections were neither uniformly overestimated nor underestimated across the cohort. However, product-specific residuals ranged from −2.97 to +3.62 log-units, reflecting significant deviations. For instance, Bavencio^®^ (Avelumab) and Imfinzi^®^ (Durvalumab) exhibited large positive residuals, with actual expenditures far exceeding projections (cost ratios of 21.5 and 1.54 in Y5, respectively), while Cotellic^®^ (Cobimetinib) and Verzenios^®^ (Abemaciclib) showed negative residuals, with actual costs well below forecasts (cost ratios of 0.03 and 0.66).

Stratified analyses revealed differences by reimbursement channel. Hospital-administered products, primarily oncology therapies, exhibited an elasticity of 0.79 (*p* = 0.055, R^2^ = 0.32), indicating less-than-proportional responsiveness. This sub-unitary elasticity likely reflects capacity constraints, delayed adoption, or competition in inpatient settings, as seen with Cotellic’s underperformance (actual Y5 expenditure €1.15 million below projections). In contrast, outpatient medicines, such as Skyrizi^®^ (Risankizumab) and Tremfya^®^ (Guselkumab), showed a higher elasticity of 1.30 (*p* = 0.003, R^2^ = 0.80), suggesting faster-than-expected cost escalation driven by rapid uptake in chronic care settings (e.g., Skyrizi’s Y5 cost ratio of 2.50).

Temporal analysis indicated that elasticity increased over time, from 0.58 in 2020 (*p* = 0.073, R^2^ = 0.39) to 0.89 in 2025 (*p* = 0.045, R^2^ = 0.41). This trend suggests progressive convergence between projected and actual expenditures as clinical adoption stabilized, and reimbursement coverage expanded. Early in the listing cycle (Y1), actual costs grew more slowly than projected, likely due to administrative bottlenecks or cautious prescribing, whereas by Y5, costs more closely aligned with forecasts, particularly for high-uptake products like Bavencio^®^ and Skyrizi^®^.

A one-sample t-test of log-ratios (log Actual/Projected costs) found no systematic bias across the cohort (mean = 0.007, *p* = 0.99), indicating that over- and under-estimations balanced out in aggregate. However, channel-specific differences emerged: hospital-administered medicines showed a slight tendency toward overestimation (mean log ratio = −0.20), while outpatient products were modestly underestimated (mean log ratio = +0.31). These directional effects were not statistically significant but align with structural differences in forecasting accuracy, with outpatient therapies (e.g., immunology) showing faster market penetration than anticipated.

## 4. Discussion

### 4.1. Accuracy of HTA Forecasts and Therapy-Specific Deviations

Our study provides a comprehensive empirical evaluation of the budgetary accuracy of HTA projections in Bulgaria by comparing expected and realized patient volumes and expenditures for medicinal products approved in 2019. Using NHIF reimbursement data through 2025, we found pronounced deviations between projected and observed outcomes, both in magnitude and direction, underscoring structural and methodological challenges in forecasting real-world adoption and financial impact within an emerging HTA system.

The divergence in patient volumes and expenditures was particularly marked among oncology and immunology therapies, where utilization frequently exceeded HTA projections by several-fold. By 2025, Bavencio^®^ (Avelumab), Skyrizi^®^ (Risankizumab), and Tremfya^®^ (Guselkumab) displayed realized-to-projected patient ratios ranging from roughly 2 to over 20, resulting in cumulative budget overshoots of €5.0 million for oncology and €17.4 million for immunology. These deviations likely stem from unanticipated indication expansions, faster-than-expected diffusion in clinical practice, and incomplete epidemiological baselines. Such findings are also consistent with international observations that BIAs often underestimate utilization for high-cost biologics and oncology products due to conservative assumptions regarding physician uptake, patient eligibility, or market entry barriers [33,42,43].

Similar discrepancies have been reported across healthcare systems. For instance, Geenen et al. [44] found that BIAs in hepatitis C significantly overestimated cost containment, while Pereira et al. [45] demonstrated underestimations of expenditure in Brazil’s enzyme replacement therapy programs due to poor modeling of treatment adherence and discontinuation. Likewise, Ravasio et al. [46] and Wong [47] showed that limited attention to regional policy heterogeneity and outdated prevalence assumptions can distort budget projections. In the Bulgarian context, such inaccuracies are magnified by the lack of robust epidemiological registries, incomplete surveillance of disease incidence, and absence of national-level data on clinical indication expansions, factors critical for valid HTA forecasting.

### 4.2. Cost Elasticity and Channel-Specific Forecasting Biases

The elasticity analysis revealed a near-proportional association between projected and realized costs across all products, suggesting that overall cost scaling was reasonable, while the model precision remained moderate. The substantial residual variation implies that current HTA forecasting approaches fail to capture product-specific diffusion dynamics. Stratified analyses showed divergent behaviors by reimbursement channel: hospital-based medicines exhibited lower elasticity, reflecting procurement delays and administrative constraints, whereas outpatient products displayed higher elasticity, indicating faster adoption once reimbursement and prescribing pathways stabilized. These findings mirror observations in other jurisdictions that hospital procurement systems often delay early adoption and inflate initial cost projections [48,49], whereas outpatient reimbursement systems tend to underestimate uptake due to more flexible prescribing and greater patient autonomy.

The temporal progression of elasticity, increasing from 0.58 in 2020 to 0.89 in 2025, suggests gradual convergence between projected and observed costs as market conditions matured and forecasting models gained calibration through real-world feedback. Early underestimation of outpatient expenditures likely reflected conservative initial assumptions and administrative lags within NHIF reimbursement approvals, a phenomenon observed across Central and Eastern Europe [24,50]. Over time, as clinical familiarity and patient identification improved, HTA forecasts became more aligned with actual uptake, indicating potential learning effects in Bulgaria’s predictive capacity.

Despite no statistically significant systematic bias overall, directional disparities emerged: hospital forecasts tended toward overestimation, while outpatient forecasts underestimated demand. These channel-specific asymmetries highlight systemic imbalances in Bulgaria’s HTA forecasting framework. In hospital settings, overestimation may stem from assuming rapid patient access that procurement and budgetary constraints later limit. Conversely, outpatient projections may fail to anticipate expanding indications, long-term adherence, and progressive case finding in chronic diseases. These observations resonate with broader European experience, where multi-indication oncology products and decentralized reimbursement systems complicate uptake prediction [29,30,51].

### 4.3. Epidemiological and Methodological Drivers of Forecasting Error

The epidemiological implications of these findings are also substantial. Forecast inaccuracies likely reflect not only methodological limitations but also data infrastructure deficits, including the absence of linked population-based registries, incomplete reporting of treated cases, and limited integration of NHIF, hospital, and morbidity datasets. As Leelahavarong [7] emphasizes, credible BIAs require clear specification of target populations and transparent epidemiological assumptions, conditions rarely met in fragmented data environments. Moreover, without accounting for epidemiologic drift (changes in disease burden, diagnostic coverage, or new indications), even well-calibrated models can quickly become obsolete.

From a policy perspective, our results reinforce the need for “living HTA” frameworks [31] that incorporate continuous epidemiological surveillance and real-time adjustment of cost projections. Bulgaria’s healthcare system, like other EU member states, faces the dual challenge of limited data infrastructure and rapidly evolving pharmaceutical markets. Addressing these issues requires the establishment of national disease registries, integration of real-world evidence into HTA updates, and inclusion of dynamic forecasting models that adjust for treatment diffusion and indication growth.

The observed heterogeneity across therapeutic areas underscores that forecasting reliability is therapy dependent. Oncology and immunology products, which dominate Bulgaria’s high-cost segment, are especially prone to underestimation of uptake due to clinical innovation cycles and evolving treatment guidelines. Similar patterns have been documented internationally for immune checkpoint inhibitors [32] and biomarker-driven oncology therapies [33], suggesting that Bulgaria’s experience reflects a broader structural limitation of traditional, static HTA approaches.

A key driver of the observed forecasting error is the reliance on static epidemiological parameters, such as fixed prevalence and incidence, that cannot capture the dynamic nature of real-world treatment pathways. Traditional BIA models assume stable patient populations with uniform treatment duration and adherence [3,9,10,11], but our findings show that these assumptions are insufficient for chronic therapies and multi-indication oncology products.

Three dimensions of treatment dynamics are particularly underrepresented in current Bulgarian HTA forecasting. First, long-term adherence and persistence vary widely across therapies: immunology products such as Skyrizi^®^ and Tremfya^®^ overshoot projections partly because patients remain on treatment longer than expected, whereas early discontinuation may explain the underuse of agents like Verzenios^®^ (Abemaciclib) [13,14]. Second, therapy switching introduces additional complexity. Rapid uptake of checkpoint inhibitors such as Bavencio^®^ and Imfinzi^®^ reflects not only incident cases but also switching from chemotherapy or earlier immunotherapies, while products like Cotellic^®^ (Cobimetinib) may be displaced by newer targeted therapies [28,29,31,43,52]. Third, forecasts often overlook high-resource utilization subgroups. Patients with advanced disease, multimorbidity, or rare conditions—such as those treated with Spinraza^®^—have highly heterogeneous treatment intensities and costs, leading to systematic underestimation when models assume uniform resource use [28,45].

### 4.4. Implications for Policy and Future HTA Practice in Bulgaria

Addressing the limitations of that static model requires transitioning from prevalence-based to dynamic cohort tracking models that integrate longitudinal patient-level data. Such systems should capture treatment initiation, continuation, switching, and discontinuation patterns in real time, enabling adaptive forecasting that responds to evolving clinical practice [53]. Linking NHIF reimbursement claims with hospital electronic health records and national disease registries would facilitate the identification of high-cost patient phenotypes and allow for risk-stratified budget projections. Furthermore, incorporating machine learning approaches to predict treatment trajectories based on patient characteristics and historical utilization patterns could materially improve forecasting accuracy, particularly for innovative therapies with limited pre-launch evidence [52,53]. International experience supports this approach. Health systems in the Netherlands, Sweden, and England have successfully implemented registry-linked HTA frameworks that continuously update budget impact models using real-world cohort data, resulting in more accurate expenditure forecasts and more equitable resource allocation [12,16,17]. Adopting similar infrastructure in Bulgaria—including mandatory post-launch data collection requirements for newly reimbursed medicines—would strengthen the evidential basis for HTA decisions and enable proactive budget management through early detection of divergence between projected and observed utilization patterns.

To improve forecasting accuracy and ensure equitable access, future HTA submissions in Bulgaria should adopt stratified budget impact models that account for patient heterogeneity. Such models should distinguish between patient subgroups defined by disease severity, treatment line, biomarker eligibility, age, comorbidity profile, and geographic region. Stratified forecasting would enable policymakers to identify which populations are most likely to drive budget overshoots, detect underserved groups with unmet needs, and design targeted managed-entry agreements or risk-sharing schemes tailored to specific patient cohorts [54,55,56,57]. This approach aligns with international best practice in equity-sensitive HTA [53,57,58] and would strengthen both the accuracy and fairness of Bulgaria’s reimbursement decisions.

Our findings have direct implications for budget governance reforms. First, dynamic contracting mechanisms, including risk-sharing agreements and pay-for-performance arrangements, could reduce projection uncertainty by linking reimbursement to actual utilization and outcomes rather than static assumptions. Second, robust epidemiologic registries are essential for improving prevalence-based forecasting, enabling more accurate estimation of target populations and uptake trajectories. Finaly, adaptive HTA frameworks that systematically incorporate real-world utilization data could allow iterative refinement of BIA assumptions, reducing the gap between projections and realized expenditures.

## 5. Limitations

This study has several limitations that should be considered when interpreting the findings. First, the absence of patient-level epidemiological data constrained the ability to explore demographic, clinical, and geographic variability in treatment uptake. Without granular data on factors such as age, sex, disease stage, comorbidities, and regional prevalence, it was not possible to determine how population characteristics or clinical profiles influenced the observed discrepancies between projected and actual utilization. For example, variations in disease severity could partially explain the higher-than-expected uptake of Bavencio and Skyrizi^®^, but these hypotheses could not be empirically tested.

The lack of stratified patient-level data also precluded subgroup analyses by key clinical and sociodemographic characteristics that are known to influence treatment uptake and costs. Stratification by disease severity, line of therapy, biomarker status, socioeconomic position, geographic region, or comorbidity burden could have revealed differential forecasting accuracy across patient subpopulations and identified vulnerable groups where projections systematically fail. Such stratified approaches are increasingly recognized as essential for equity-sensitive HTA and for identifying which patient cohorts drive budget deviations.

Second, the exclusion of nusinersen (Spinraza^®^) due to missing HTA budgetary projection data likely introduced bias in evaluating rare-disease therapies, which have distinct epidemiological and financial profiles. Such conditions often involve low incidence, diagnostic delays, and highly variable eligibility criteria, all of which make budget forecasting inherently uncertain. Consequently, omitting Spinraza^®^ may have understated the heterogeneity and fiscal impact of rare-disease medicines.

Third, the use of aggregate median monthly values for patient counts and costs may have masked seasonal or episodic variations in utilization. Hospital-administered oncology products frequently display cyclic demand linked to treatment regimens or hospital capacity, while outpatient therapies may vary with patient adherence or administrative delays in NHIF reporting. This aggregation, while necessary for consistency, may thus have concealed short-term utilization spikes or gaps that carry epidemiological significance.

Fourth, the analysis focused solely on the 2019 HTA cohort, limiting generalizability to other assessment years or therapeutic domains. The accuracy of projections may have evolved alongside methodological refinements at the National Council on Prices and Reimbursement and as real-world data availability improved. Moreover, this single-cohort approach does not capture epidemiologic drift—changes in disease incidence, diagnostic criteria, or clinical indications that alter patient eligibility over time. For instance, the rapid expansion of Bavencio^®^ and Skyrizi^®^ usage may reflect new indications or improved disease recognition rather than forecasting error per se.

Fifth, the study relied exclusively on NHIF administrative data, which, while comprehensive, are subject to coding inconsistencies, reporting delays, and potential underreporting—particularly for high-cost hospital therapies or outpatient dispensing. These issues may have introduced misclassification or measurement error. Additionally, patients treated outside NHIF reimbursement (e.g., in clinical trials, compassionate use, or special funds) were not captured, potentially underestimating real utilization.

Sixth, additional constraints include the single-country focus, which limits external validity given health system heterogeneity across nations; the limited number of post-2019 innovations, which may reduce generalizability to rapidly evolving therapeutic areas; potential temporal confounding from data lag and pricing adjustments over the five-year period; and the absence of manufacturer-level variables (e.g., marketing intensity, launch timing), which limits attribution of observed deviations to systemic versus product-specific factors.

Finally, epidemiological and market factors external to the HTA process—such as shifts in clinical guidelines, competition from newer drugs, or pricing renegotiations—were not explicitly modeled but likely influenced real-world deviations. The retrospective design also limits insight into the forecasting context of 2019, when projections were made using limited pre-launch data and without the benefit of robust national disease registries.

Future research should examine the determinants of uptake deviations, including clinical guideline updates, off-label use, and patient access barriers. Cross-country comparisons could clarify whether Bulgaria’s forecasting challenges are system-specific or part of broader limitations in HTA practice [44]. Finally, adopting machine learning and adaptive forecasting approaches may enhance the accuracy of budget impact projections, particularly for high-cost biologics and oncology therapies [45].

## 6. Conclusions

HTA budget-impact projections in Bulgaria diverged substantially from real-world outcomes, particularly for high-cost oncology and immunology therapies. These errors were driven mainly by utilization rather than price, reflecting gaps in epidemiologic data and limited real-time monitoring of treatment patterns. Strengthening national registries and establishing routine real-world data pipelines that support dynamic cohort tracking would improve forecast accuracy and support more equitable and sustainable budget planning.

To address these challenges, future BIAs should incorporate explicit patient-flow modeling and real-world persistence data and should provide stratified forecasts that reflect clinically and economically relevant patient subgroups such as those defined by disease severity, treatment line, and comorbidity burden. These improvements must be supported by mandatory post-launch surveillance for newly reimbursed products and by the adoption of “living” HTA processes in which budget-impact projections are updated regularly as new data accumulate. Embedding these requirements within the procedures of the National Council on Prices and Reimbursement would materially strengthen Bulgaria’s HTA framework, enhance the predictive validity of BIAs, and improve the stewardship of NHIF resources while ensuring equitable access to innovative therapies.

## Figures and Tables

**Table 1:** Medicinal products approved after HTA evaluation in Bulgaria in 2019 with corresponding indications.

Market Name	INN	Indication	Funding Channel
Alunbrig^®^	Brigatinib	Non-small cell lung cancer (ALK-positive NSCLC)	Hospital
Bavencio^®^	Avelumab	Merkel cell carcinoma (MCC, metastatic skin cancer)	Hospital
Cotellic^®^	Cobimetinib	Metastatic melanoma with BRAF V600 mutation (in combination with vemurafenib)	Hospital
Hemlibra^®^	Emicizumab	Hemophilia A	Outpatient
Imfinzi^®^	Durvalumab	Non-small cell lung cancer (NSCLC) and small cell lung cancer (SCLC)	Hospital
Imnovid^®^	Pomalidomide	Multiple myeloma	Hospital
Juluca^®^	Dolutegravir/Rilpivirine	HIV-1 infection	HIV program
Latuda^®^	Lurasidone	Schizophrenia and bipolar disorder	Outpatient
Skyrizi^®^	Risankizumab	Plaque psoriasis and psoriatic arthritis	Outpatient
Spinraza^®^	Nusinersen	Spinal muscular atrophy (SMA)	Outpatient
Tremfya^®^	Guselkumab	Plaque psoriasis	Outpatient

**Table 2:** HTA projections vs. annualized actual treated patients and observed-to-projected ratios (2019 HTA cohort; 2020 & 2025).

Market Name	INN	Projected (Y1|Y5)	Actual (Y1|Y5)	Ratio Y1 ^a^ (Min–Med–Max)	Ratio Y5 ^a^ (Min–Med–Max)	Cost Ratio ^b^ (Y1|Y5)	Cost Gap ^c^ (€) (Y1|Y5)
Alunbrig^®^	Brigatinib	4.6|11	36|198	0.22–0.65–1.09	1.27–1.50–1.64	0.66|1.52	−62,606|179,497
Bavencio^®^	Avelumab	4|8	60|2040	0.50–1.25–1.75	19.4–21.3–22.2	1.10|21.5	14,117|7,642,580
Cotellic^®^	Cobimetinib	43|45	60|12	0.07–0.12–0.23	0.02–0.02–0.04	0.14|0.03	−1,257,502|−1,151,943
Hemlibra^®^	Emicizumab	16|38	24|336	0.13–0.13–0.13	0.63–0.74–0.92	0.12|0.76	−1,394,694|−1,264,157
Imfinzi^®^	Durvalumab	46|144	198|2688	0.22–0.36–0.41	1.48–1.55–1.62	0.36|1.54	−757,149|4,476,511
Imnovid^®^	Pomalidomide	25|35	30|174	0.08–0.10–0.12	0.31–0.41–0.91	0.10|0.72	−468,769|−154,140
Latuda^®^	Lurasidone	270|690	1416|8064	0.22–0.44–0.55	0.95–0.97–1.01	0.41|1.06	−66,716|26,920
Skyrizi^®^	Risankizumab	45|400	558|11,976	0.71–1.03–1.58	2.13–2.50–2.80	1.04|2.50	46,091|11,140,253
Spinraza^®^	Nusinersen	—|—	66|132	—	—	—|—	—|—
Tremfya^®^	Guselkumab	110|570	288|13,296	0.21–0.22–0.41	1.68–1.94–2.23	0.24|1.94	−1,101,437|6,275,740
Verzenios^®^	Abemaciclib	167|927	246|7464	0.02–0.12–0.29	0.64–0.67–0.74	0.15|0.66	−1,926,427|−6,036,965

^a^ Ratios indicate realized-to-projected patient volumes (min–median–max across observed months). ^b^ Cost ratio = actual|projected expenditure. ^c^ Cost gap = actual − projected (in euros). Y1 year refers to the first HTA projection horizon (2020, except Alunbrig 2021).

## Data Availability

All datasets are distributed under the Creative Commons Attribution (CC BY 4.0) license and are openly accessible for validation and reuse. The datasets generated and analyzed during the current study are publicly available in the Zenodo repository:
Kostadinov, K. (2025). *NHIF Bulgaria: Expenditures and Patient Counts for Antineoplastic and Coagulopathy Medicines in Hospital Treatment* [Data set]. Zenodo. https://doi.org/10.5281/zenodo.17428045 ([38], accessed on 27 October 2025). Kostadinov, K.; Shopova, M.; Petkov, P.; Ovchinnikov, E.; Raycheva, R. (2025). *NHIF Bulgaria: Expenditures and Patient Counts for Home Treatment Medicines and Medical Products 2021–2024 (by Region, NHIF Code, and ICD Code)* [Data set]. Zenodo. https://doi.org/10.5281/zenodo.17428116 ([39], accessed on 27 October 2025).

## Abbreviations

The following abbreviations are used in this manuscript:

ATC	Anatomical Therapeutic Chemical (classification)
BGN	Bulgarian lev
BIA	Budget Impact Analysis
EHDS	European Health Data Space
EU	European Union
EUR	Euro
FAIR	Findable, Accessible, Interoperable, Reusable (data principles)
HIV	Human Immunodeficiency Virus
HTA	Health Technology Assessment
ICD-10	International Classification of Diseases, 10th Revision
INN	International Nonproprietary Name
ISPOR	International Society for Pharmacoeconomics and Outcomes Research
MCC	Merkel cell carcinoma
NCPR	National Council on Prices and Reimbursement (of Medicinal Products)
NCPRMP	National Council on Prices and Reimbursement of Medicinal Products
NHIF	National Health Insurance Fund
NSCLC	Non-small cell lung cancer
PDL	Positive Drug List
RWD	Real-world data
SCLC	Small cell lung cancer
SMA	Spinal muscular atrophy
